# Comparative Clinical Study on Periodontal Health Status and Early Diagnosis of Periodontal Diseases Quantified through Clinical Periodontal Indices on a Group of Children and Adolescents with and without Cardiovascular Diseases

**DOI:** 10.3390/pediatric16010001

**Published:** 2023-12-26

**Authors:** Oana Chipirliu, Marian Viorel Crăciun, Madalina Nicoleta Matei

**Affiliations:** 1 Research Centre in the Medical-Pharmaceutical Field, Faculty of Medicine and Pharmacy, Dunarea de Jos University of Galati, 47 Domneasca Str., 800181 Galati, Romania; marian.craciun@ugal.ro (M.V.C.); madalina.matei@ugal.ro (M.N.M.); 2 Research Centre in the Faculty of Automation, Computers, Electrical and Electronics Engineering, Dunarea de Jos University of Galati, 111 Domneasca Str., 800181 Galati, Romania

**Keywords:** inflammatory condition, periodontal disease, children and teenagers, cardiovascular diseases, plaque index, gingival index, gingival bleeding index, microbiome, predisposing factors, vicious

## Abstract

It is well known that bacterial plaque is the main etiological factor that causes the appearance of periodontal diseases and carious disease. Periodontal diseases can affect children and adolescents and are manifested in the form of gingivitis, but also the early form of chronic periodontitis as well as aggressive marginal periodontitis associated with local or general factors. Early periodontitis is frequently undiagnosed by clinicians due to a lack of knowledge of the specific symptoms. Certain systemic diseases, such as cardiovascular diseases, can create favorable conditions for the appearance and progression of severe manifestations of periodontal disease; also, recent research highlights that individuals with periodontal disease present an increased risk of developing cardiovascular diseases. Children with congenital or acquired cardiovascular diseases are at increased risk for complications resulting from the growth of microorganisms in the oral cavity, presenting a risk of infective endocarditis. The specific aim was to highlight the existing differences between the periodontal health of children with cardiovascular diseases and that of children without these diseases. The analyzed group included 124 patients, represented by children and adolescents, aged between 7 and 17 years, who were divided into four subgroups depending on the presence or absence of cardiovascular diseases and periodontal disease. A specialized clinical examination was performed for each patient, and periodontal clinical parameters were quantified (plaque index, gingival bleeding index, gingival index, community periodontal index of treatment needs) and associated with the diagnosis of general condition. Patients diagnosed with periodontal disease underwent specialized treatment and were called to a control visit 3 months after treatment. Statistical analysis showed significant differences between subgroups with much higher values of clinical parameters for patients with cardiovascular disease. Also, the response to the treatment was better in the case of patients in the control subgroup without cardiovascular diseases. The present study highlighted the interaction of three factors in the progression of periodontal diseases: subgingival microbiota, immune system response and environmental factors.

## 1. Introduction

As mentioned by the World Health Organization, oral diseases, including periodontal disease, constitute a significant and essential part of the overall health of the individual [1,2].

It is well known that bacterial plaque is the main etiological factor that causes the appearance of periodontal diseases and carious disease [3]. Periodontal diseases can affect children and adolescents and are manifested in the form of gingivitis, but also the early form of chronic periodontitis as well as aggressive marginal periodontitis associated with local or general factors. Early periodontitis is frequently undiagnosed by clinicians due to a lack of knowledge of the specific symptomatology [3]. Certain systemic conditions, such as cardiovascular diseases, diabetes or obesity, can create favorable conditions for the appearance and progression of severe periodontal disease manifestations, rampant evolutions of various complicated forms of these conditions and poor responses of the host organism to specialized treatment [3,4].

Also, recent research highlights that individuals with periodontal disease present an increased risk of developing cardiovascular diseases. Local infection caused by periodontal disease could compromise the levels of systemic inflammatory mediators, thus favoring the mechanisms of atherosclerosis and coronary complications of this condition [3,5].

Children with congenital or acquired cardiovascular diseases are at increased risk for complications resulting from the growth of microorganisms in the oral cavity, presenting a risk of infective endocarditis [6,7].

Compromised general health can cause damage to the immune system and the appearance of severe forms of periodontal disease at young ages. Gingival diseases are common in children and young people and, in the absence of appropriate treatment, they can evolve into forms of deep periodontal damage of the marginal periodontium [8,9].

The periodontal health of children and adolescents should be assessed at every oral examination. Oral hygiene should be assessed through plaque indices, and the use of bacterial plaque revealers can provide a way to monitor and document oral hygiene practices [8].

The appearance of the first signs of periodontal damage, manifested by edema, inflammation and gingival bleeding, can be observed during the specialized clinical examination of the oral cavity by inspection, palpation, measurement of periodontal parameters and quantification of oral hygiene and gingival inflammation indices.

Adolescence is a transitional stage of physical and mental human development that occurs between puberty and maturity. Many diseases diagnosed in adulthood have their origin in adolescence and are related to lifestyle. For example, smoking, unhealthy diet, premature stress and lack of physical exercise cause progressive problems that lead to chronic diseases or premature death. Chronic periodontitis can also have its beginnings in adolescence and can later, in adulthood, lead to tooth loss as well as the association of oral cavity diseases with systemic diseases [9].

In the previously stated context, the main purpose of this study was to quantify the degree of oral hygiene and the clinical manifestations of periodontal disease or periodontal health, recorded through the periodontal clinical examination and the hygiene and periodontal inflammation indices for the subjects included in the study. The effect of local periodontal treatment on clinical periodontal parameters was also followed, comparing patients with periodontal diseases and patients without a diagnosis of affected general condition.

Considering the purpose of the study, the present research starts from the hypothesis that the use of new techniques regarding the correlations between cardiovascular diseases and periodontal disease, in addition to those already known, insufficiently analyzed in the specialized literature, will have beneficial effects in medical practice through a better evaluation of these conditions, which are seen as two diseases that can influence each other from the point of view of the manifested symptomatology, as well as beneficial effects in the treatment applied to the patients as well as in the host’s response to the applied treatment.

## 2. Materials and Methods

The patients included in the batch were selected from the patients admitted to the cardiology department of the Emergency Clinical Hospital for Children “St. Ioan” from Galati and among the patients of a private dental practice in the city of Galati. The study was conducted over a period of 12 months, between May 2022 and April 2023. A number of 124 patients, represented by children and adolescents, aged between 7 and 17 years, were included in the batch. Informed consent was obtained, and each participant (legal representative) voluntarily signed the informed consent and clinical trial participation form. Patients were informed about the study in which they were invited to participate through an information form and detailed explanations about the research procedures and aims. Participants agreed to the necessary clinical and paraclinical investigations by signing an informed consent form to participate in the study.

The selection of patients was carried out respecting the following types of criteria:

1. Criteria for inclusion in the study group of patients: age between 7 and 17 years; the presence of at least 6 dental units, represented by young permanent teeth, erupted on the arch; patients who have not benefited from professional sanitation in the last 3 months; patients who received a diagnosis of cardiovascular disease confirmed by complementary investigations and required specialized care (sublot A, B); children and adolescents with a good general condition, without a diagnosis of impaired general condition (sublot C, D);
2. Exclusion criteria from the study group of patients: age greater than 17 years or less than 7 years; patients who have followed in the last 3 months an antimicrobial and anti-inflammatory treatment with antibiotics; patients who wore fixed orthodontic appliances; patients who have suffered from infective endocarditis and require treatment with prophylactic antibiotics for periodontal examination and specialized procedures; patients who had severe cardiovascular diseases and had an unstable general state of health; patients with allergic terrain; patients whose data were inaccessible or incomplete or who did not agree to participate in the study.

The required number of patients for the evaluation of clinical parameters for the early diagnosis of periodontal diseases was established by applying the formula:

$$n=\frac{1}{\left(1-f\right)}\times \frac{2(z\alpha +z\beta ){}^{2}\times p\times \left(1-P\right)}{\left(P0-P1\right)}$$

where:

*P*0—the proportion of patients with early gingival diseases (researchers demonstrate approximately 14%).
*P*1—the proportion of patients with early gingival diseases diagnosed at the first specialist consultation.
*zα*—tabular value. When “*α*”—the significance threshold—is 5%, then the coefficient *zα* = 1.96 *zβ*—tabular value. Când “*β*”—the statistical power of the comparison is 5.0%,
*f*—Proportion of subjects expected to drop out of the study for reasons other than the effect investigated.

Entering the data into the formula, we obtained *n* = 62 (62 patients with periodontal disease and 62 without periodontal disease); which were divided according to the general condition into subgroups of 31 patients.

A number of 164 patients were enrolled in the initial group, of which 40 were excluded as they did not meet the inclusion criteria: a number of 31 people did not want to participate in this study (*n* = 31), 3 patients had followed antibiotic and anti-inflammatory treatment in the last 3 months (*n* = 3), 3 patients were wearers of orthodontic appliances (*n* = 3), 2 patients suffered from a severe cardiovascular disease (*n* = 2) and 1 patient had allergic terrain (*n* = 1).

The total group of examined patients consisted of 124 patients, who were divided into four subgroups as follows:

- Subgroup A—consisting of 31 (25%) patients with cardiovascular diseases and periodontal diseases;
- Subgroup B—consisting of 31 (25%) patients with cardiovascular diseases and without periodontal diseases;
- Subgroup C—consisting of 31 (25%) patients without cardiovascular diseases but with periodontal diseases;
- Subgroup D—consisting of 31 (25%) patients without cardiovascular diseases and without periodontal damage.

Periodontal clinical examination: Assessment of the odonto-periodontal status was carried out through a general, local clinical examination and paraclinical investigations. All patients in these groups were given observation sheets that included anamnesis and clinical examination.

The clinical examination included three main stages:

- Anamnesis—data related to periodontal damage were collected from the reports of the patients and their families.
- The objective clinical examination—for the selection of local information and the preparation of a presumptive diagnosis.
- Complementary examinations—which helped to establish the diagnosis and to choose some prophylaxis or treatment measures (panoramic radiographs).

The anamnesis helped us to select general information related to:

- Identity, age, sex;
- Eating habits;
- The existence of some vicious habits (smoking, alcohol consumption, bruxism);
- The reasons for presenting to the doctor;
- The hereditary-collateral information related to the diagnosis of the general condition (presence of cardiovascular diseases in the family) or local condition (presence of periodontal diseases in the family);
- History of oral and periodontal diseases.

The objective clinical examination was performed by inspection, palpation and percussion, and the features of the superficial and deep marginal periodontium were followed.

During the clinical examination of the superficial marginal periodontium, the following were observed:

- Appearance of interdental papillae;
- The position of the free gingival margin and fixed gingiva;
- The presence of changes in color, texture, volume, consistency and adhesion to the underlying planes.

The existence of gingival recessions was recorded by measuring in millimeters on all sides of the tooth from the level of the anatomical package to the level of the free gingival margin. The information was recorded in the observation sheet and in the periodontogram for each patient.

**Figure 1:**
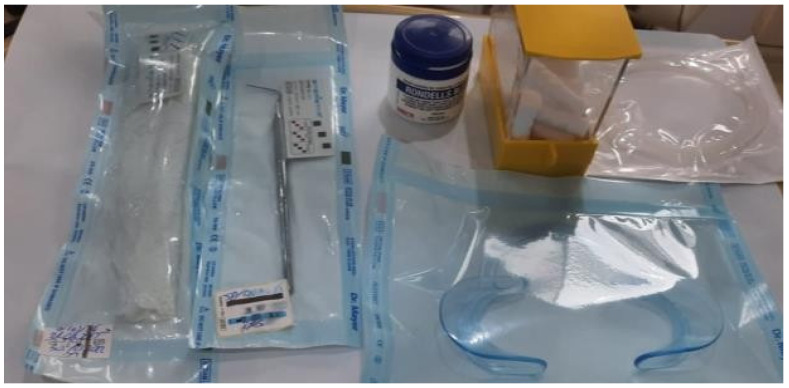
Instrumentation and materials used for patient consultation.

**Figure 2:**
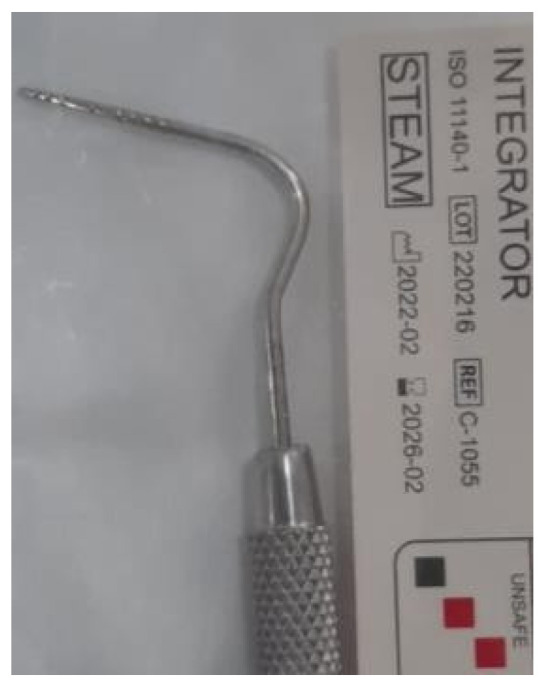
Graduated periodontal probe used for periodontal probing.

For the clinical examination of the deep periodontium, the classic consultation kit (mirror, probe, tweezers, spatula) and Williams-type buttoned periodontal probe were used, with the active part marked from millimeter to millimeter, up to 3 mm, then to 5 mm and then 7 mm and again from milliliter to milliliters up to 10 mm. (Figure 1 and Figure 2) Williams periodontal probes were invented in 1936 and quickly became the model for all future periodontal probes. This probe has a fine gradation of the active part and the buttoned tip in order to not damage the periodontium during the examination procedures [10,11,12].

The survey was carried out in six points for each tooth: mesio-vestibular, centro-vestibular, disto-vestibular, mesio-palatal (or lingual), centro-palatal (or lingual) and disto-palatal (or lingual). We used a probing force not exceeding 0.2–0.25 N [8,13].

The dentition was divided into six sextants: upper right (17 to 14), upper anterior (13 to 23), upper left (24 to 27), lower right (47 to 44), lower anterior (43 to 33), lower left (34 to 37) [12].

For the age range 7–12 years, the simplified periodontal screening was used, analyzing only six index teeth: right maxillary central incisor, right and left maxillary first molar, left mandibular central incisor, right and left mandibular first molar (1.1, 1.6, 2.6, 3.1, 3.6, 4.6) [14,15,16].

**Figure 3:**
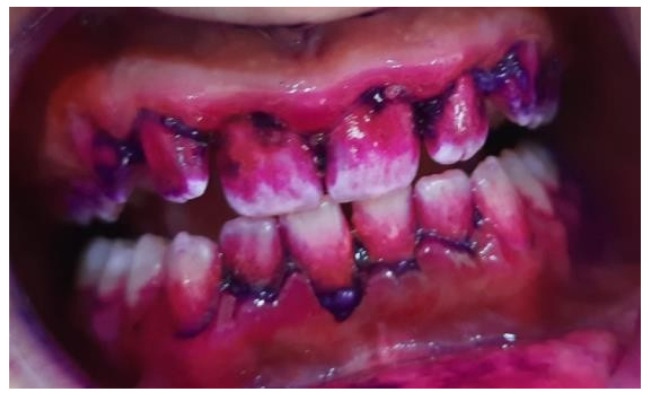
Staining of bacterial plaque using RONDELLS BLUE tablets, Directa, 2-tone stain showing areas of older plaque (>3 days) and new plaque—red/pink, patient 14 years, lot A.

**Figure 4:**
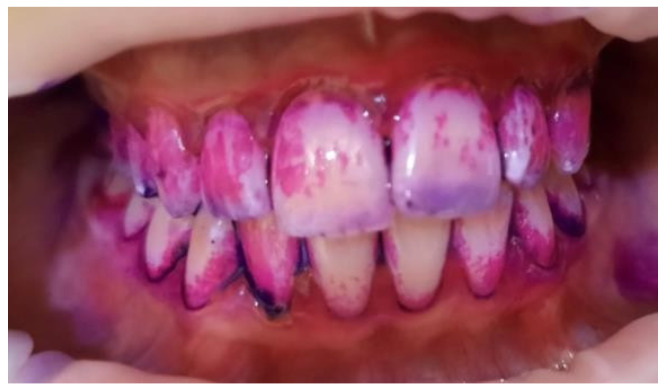
Bacterial plaque staining using RONDELLS BLUE tablets, Directa, 2-tone stain showing areas of older plaque (>3 days) and new plaque—red/pink, 16-year-old patient, batch C.

Plaque index (IP) values were calculated using the formula: the number of dental surfaces with plaque divided by the total number of dental arch surfaces, and the result multiplied by 100 (Figure 3 and Figure 4).

$$\mathrm{Plaque}\;\mathrm{index}\left(\mathrm{IP}\right)\;\mathrm{values}=\frac{\left(\mathrm{number}\;\mathrm{of}\;\mathrm{dental}\;\mathrm{surfaces}\;\mathrm{with}\;\mathrm{plaque}\right)}{\left(\mathrm{total}\;\mathrm{number}\;\mathrm{of}\;\mathrm{dental}\;\mathrm{arch}\;\mathrm{surfaces}\right)}\times 100$$

The following information was used to interpret the plaque index (IP) values:

IP ≤ 10% Effective oral hygiene.
IP ≤ 20% Good oral hygiene.
IP > 20% Unsatisfactory oral hygiene.

The gingival bleeding index (BOP—bleeding on probing) was used to assess the degree of inflammation: if bleeding occurs within 10–15 s after probing, the respective site receives a positive score. The bleeding index value was calculated by adding the number of bleeding surfaces divided by the total number of dental surfaces examined and multiplied by 100 [17,18].

$$\mathrm{BOP}=\frac{\mathrm{number}\;\mathrm{of}\;\mathrm{bleeding}\;\mathrm{surfaces}}{\mathrm{number}\;\mathrm{of}\;\mathrm{dental}\;\mathrm{surfaces}\;\mathrm{examined}}\times 100$$

The gingival index (Loe and Silness) (IG) was assessed using data collected by gentle palpation inspection and periodontal survey [17,18].

Interpretation of gingival index values:

0 = Gum with normal appearance;
1 = Gum with mild inflammation, discrete color changes, discrete edema, lack of bleeding on probing;
2 = Medium inflammation, congestive, edema, bleeding on probing;
3 = Advanced inflammation, congestion, stasis, ulcerations, spontaneous bleeding.

Community periodontal index of treatment needs (CPITN) was used to quantify the need for treatment of the groups included in the study. The CPITN was introduced by the World Health Organization (WHO) in 1977 and used to assess the prevalence of chronic inflammation caused by periodontal disease in numerous communities. Recently, it was renamed as the community periodontal index (CPI) [17].

By counting this index, the parameters of periodontal damage and the need for treatment were recorded for each of the six sextants.

Interpretation of the registered periodontal status:

0 = Healthy periodontium.
1 = Bleeding seen directly on probing.
2 = Supra/subgingival tartar and pockets below 3.5 mm, iatrogenic irritation.
3 = Bags of 4–5.5 mm.
4 = Bags > 6 mm [17].

**Figure 5:**
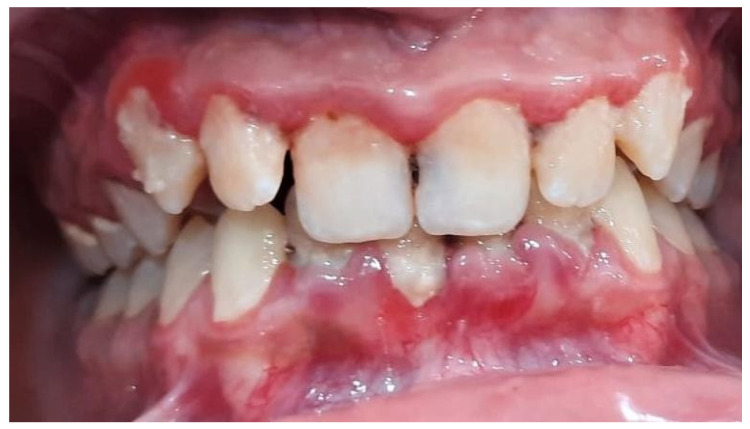
Patient 14 years old, RVG, IP = 90%, BOP = 86%, IG = 4, CPITN = 3, SP val med = 3.5 mm.

**Figure 6:**
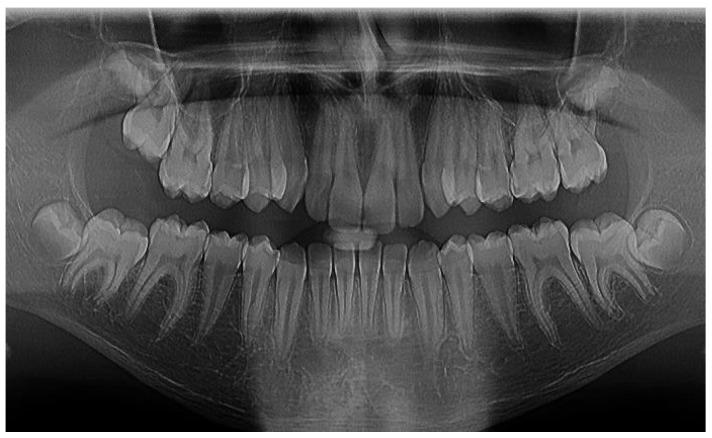
OPG, patient 14 years RVG.

Based on the recorded values, the analyzed patients were included in one of the following periodontal treatment classes:

Code 0 = does not require periodontal treatment, periodontal health (code 0).
Code I = indications for improving oral hygiene, professional advice (code 1).
Code II, III = indications for improving oral hygiene, professional instillation, professional descaling, scaling and root planing, antibacterial treatment, removal of microirritation factors (code 2 and 3).
Code IV = includes the stages for codes I + II + III and the completion of complex surgical periodontal treatment, functional occlusal rebalancing, local and general biostimulation (code 4) (Figure 5 and Figure 6) [17].

The data collected at the clinical examination were recorded in the periodontogram, where we received a graphic representation of the periodontal status. To assess the risk of developing periodontal diseases, the patients’ data were included in the periodontal risk assessment program depending on the degree of hygiene and the presence of general diseases.

A panoramic radiograph was made for each patient for a diagnosis of certainty and to complete the information collected during the clinical examination.

The study protocol covered the following stages:

1. The initial screening visit—which included anamnesis, general clinical examination, periodontal clinical examination;
2. Complementary investigations—radiological examinations;
3. Instruction on maintaining oral hygiene—mastering the correct brushing techniques and the use of hygiene aids, demonstrations on correct brushing for all patients;
4. The stage of initial periodontal treatment applied to participants from subgroups A and C;
5. Evaluation stage 3 months after initial periodontal treatment—clinical examination, recording of periodontal parameters for subgroups with periodontal damage.

Treatments and manipulations were performed as follows.

Patients from subgroups B and D, as well as their families, were trained on maintaining oral hygiene through the correctness of brushing techniques and the use of oral care aids (dental floss, mouthwash, interdental brushes, mouthwash).

The patients in subgroups A and C were subjected to a periodontal treatment protocol (initial stage) that consisted of: motivation to maintain periodontal health through rigorous oral hygiene instruction (learning the correct brushing techniques and use of oral care aids), descaling supragingival (ultrasonic/manual where needed), airflow (where necessary), professional brushing to create favorable local conditions for healing and maintaining good gingival health. Recommendation of mouthwashes with chlorhexidine 0.12% for three weeks, for severe forms of gingival inflammation, as well as local applications, in the office, of antiseptic substances (chlorhexidine 0.20%, gel with chlorhexidine 1%, glycosite gel, gel with metronidazole), were given. For children aged between 7 and 12 years, professional brushing, carried out in the office, was sufficient to remove the microbial factor from the oral cavity.

For all patients, the bacterial plaque was revealed and professional brushing was performed. Periodontally healthy patients (subgroups B and D) were assessed only at the initial visit, where the diagnosis of periodontal health was established. Patients from the subgroups with gingival–periodontal diseases (subgroups A, C) were examined at the initial visit and three months post-therapy.

The data obtained from the clinical examinations and through the analysis of the computerized records were entered into the table using the Microsoft Excel 2011 program for better centralization and to form the database necessary for the statistical analysis. In our study, descriptive statistics (percentage, mean, frequency and standard deviation) were calculated as the first step of data analysis. The data set was subsequently analyzed using statistical tests: Welch’s *t*-test, Cohen’s d-statistic and the graphical method, using the R programming language. The applied null hypothesis was that there were no statistically significant differences between the means of the parameters for the four subgroups [19,20,21,22].

Welch’s *t*-test, which measures the difference between two groups, comparing their means without assuming the populations from which the two groups have come from, have the same statistical variance (variance = square of the standard deviation; measures the distribution of values around the mean or away from the mean), as does the Student’s *t*-test [19,20].

Welch’s test was used to compare subplots A vs. C and B vs. D, as well as A, C, vs. B, D values. If the averages of the analyzed subgroups differed significantly, at a level of significance given by values of *p* < 0.05, and for *p* ≤ 0.001, it was considered highly statistically significant for the compared values. For the graphical representation of the test, if the value of *p* was very small (close to 0), **** was awarded, and if the values were closer to 0.001, *** was awarded [19,20] (Table 1).

Cohen’s test measures the size of the impact of a parameter, quantifying the difference between the compared groups. One of the most common measures of effect size used for statistical analysis was Cohen’s d, which is calculated as follows: [21].

d = (x1 − x2)/√(s1^2^ + s2^2^)/2

where x1, x2 is the average of sublots A and C, respectively, or average of sublots B and D.

s1^2^, s2^2^ is the variation in sublot A and C, respectively, or variation in sublots B and D.

The values of d can present the following variations, which indicate information on the averages of the analyzed sublots:

- d of 0.5 indicates that the means of the two subplots differ by 0.5 standard deviations;
- d of 1 indicates that group means differ by 1 standard deviation;
- d of 2 indicates that group means differ by 2 standard deviations.

Cohen’s d can also be interpreted as follows: an effect size of 0.5 means that the value of an average person in group 1 is 0.5 standard deviations above the average person in group 2.

We used the following rule of thumb when interpreting Cohen’s d:

A value < 0.2 represents a very small effect size.
A value between 0.2≤ and <0.5 represents a small effect size.
A value between 0.5≤ and <0.8 represents a medium effect size.
A value 0.8≤ represents a large effect size [21,22].

The results are presented as arithmetic means ± standard deviation for quantitative variables.

The initial batch of patients consisted of 164 patients, of which 40 patients were excluded because they did not meet the conditions for inclusion in the batch. The remaining group consisted of 124 patients, divided into four subgroups: A, B, C, D. For each sublot, the general condition parameters were compared: weight, height, body mass index (BMI), cardiovascular diagnosis, heredo-collateral antecedents of cardiovascular diseases. Epidemiological data were also analyzed, such as age, gender, environment of origin (U/R), vicious habits (YES/NO). For each subgroup, periodontal and oral hygiene indices were recorded at the time of the initial consultation, marked with T1. In the case of the subgroups diagnosed with periodontal disease, the clinical periodontal parameters were recorded at the time of T1 and 3 months after the application of the specialized treatment, denoted by T2.

The statistical analysis highlighted statistically significant differences between the analyzed groups for the general and epidemiological data, which are presented numerically and graphically. The tables and figures within this section offer a brief summary of how the IP, BOP, IG, DP and CPITN indices are distributed at the T1 and T2 time points in the conducted study, focusing on the essential features among the subgroups of the analyzed population.

## 3. Results

In the entire group analyzed, the average age was 14 years, while in the subgroups with periodontal diseases, the average age was 13.5 years. In the subgroups diagnosed with periodontal health, the mean age was 14.5 years. Between the four subgroups, no significant difference is observed in terms of the mean age of the included patients (Table 2).

Regarding distribution by gender, in the total group, the female gender dominated with an average of 54.83%. In the subplots with periodontal diagnosis, the male gender was predominant at 54.83% (subplot A) and 61.29% (subplot C). In the subgroups with a diagnosis of periodontal health, the female gender was predominant at 74.19% and 61.29% (subgroups B and D). A statistically significant difference is observed regarding the distribution by gender at the level of the subgroups with periodontal damage compared to the subgroups diagnosed with periodontal health.

The environment of origin, at the level of the whole lot, was most represented by the urban environment, it being much easier to reach for consultations and treatment, at 74.17% (number 90), where the value was only 26.82% (number 34) for the rural environment. The most numerous patients from rural areas were registered in subgroup A, with 10 patients representing 32.25%, and the smallest number was found in subgroup D, with 7 patients representing 25.58%. The urban environment can represent a predisposing factor for the development of periodontal and cardiovascular diseases due to premature exposure to numerous risk factors, age-inappropriate diet, stress, sedentary lifestyle and poor oral hygiene.

For vicious habits, at the level of the total group of 124 patients, 21 (16.92%) patients with vicious habits were identified: 1 patient with bruxism (0.80%), 15 smoking patients (12.96%) and 5 patients with oral breathing (12.96%). Smoking was confirmed as a predisposing factor for cardiovascular diseases and periodontal disease at the level of subgroup A, with the most smokers being identified—a number of eight patients, representing 25.80% of the total number of patients—compared to the rest of the subgroups, where the number of smokers is less high: at the level of subgroups B, D, there are three (9.67%) smokers and two (6.45%) smokers, respectively (Table 3).

Regarding the heredo-collateral antecedents of periodontal diseases (PD), at the level of the total group, a number of 44 patients with first-degree relatives, presenting periodontal diseases (35.48%), was counted. A large number can be found at the level of the groups with periodontal diseases, where 19/61.29% patients were identified for subgroup A and 11/35.48% patients for subgroup C, compared to the groups diagnosed with periodontal health, where the number is much lower at 6/19.35% patients for subgroup B and 8/25.80% patients for subgroup D. Statistically significant differences between the subgroups are observed for the heredo-collateral antecedents of periodontal diseases. The genetic predisposing factor for the development of periodontal diseases is confirmed.

Also, for the heredo-collateral history of cardiovascular diseases (CDs), at the level of the total group, 36/29.03% of the patients had a family history of cardiovascular diseases; per group, their distribution was as follows: 13/41.93% patients in subgroup A, 11/35.48% patients in subgroup B, 7/22.58% patients in subgroup C and 5/16.12% patients in subgroup D. There are statistically significant differences between the subgroups with cardiovascular diseases compared to the subgroups with good general condition regarding heredo-collateral antecedents. The genetic factor predisposing to cardiovascular diseases is confirmed (Table 4).

Regarding the diagnosis of cardiovascular diseases, at the level of the total group of 124 patients, their distribution was as follows: arrhythmia = 3/2.41% patients, sinus bradycardia = 6/4.83% patients, hypertrophic cardiomyopathy = 2/1.61% patients, DSA = 8/6.45% patients, DSV = 12/9.67% patients, HTN = 13/10.48 patients, heart failure = 9/7.25% patients, PCA = 1/0.80 patients and tachycardia = 8/6.45% patients; 62/50.00% of patients did not present. Congenital cardiovascular diseases, as well as acquired ones, were identified. The main discharge diagnosis was recorded for each patient.

Regarding the body mass index (BMI) at the level of the total group, 104/83.87% had normal body mass and 20/16.13% of patients had modified body mass. Overweight patients could be found in subgroups A, B: 3/9.67% overweight patients and 3/9.67% severely overweight patients (subgroup A), and 2/6.45% overweight patients and 1/3.22% severely overweight patients (subgroup B). As can be seen, a total of 4/3.22% patients were identified as severely overweight according to the height–weight parameters, with 3/9.67% patients and 1/3.22% patient identified at the level of subgroup A and subgroup B, respectively. There are statistically significant differences for the modified BMI across subgroups with cardiovascular disease.

Significant differences were observed between the values of the clinical parameters, comparing the subgroups with a diagnosis of periodontal damage and the subgroups with a diagnosis of periodontal health. Also, different values were recorded between periodontal clinical parameters for subgroups A, C at the initial consultation T1 and at T2, after the application of the initial periodontal treatment.

After the application of the periodontal treatment specific to the initial phase, a significant evolution of the clinical parameters toward the state of periodontal health was observed. The results are more obvious in the case of patients from subgroup C, with periodontal damage, but with good general condition, where PI T1 = 50.94% compared to PI T2 = 5.90%; the initial GI was 1.48 and, after treatment, it was considerably reduced to 0.03; the BOP at T1 = 38.81% and reduced at T2 to values of 2.35%; the probing depth DPT1 = 2.55 mm and, later, 1.70 mm; and the CPITN T1 = 2.29 and, after treatment, the CPITN T2 = 0.16 (Table 5).

A graphical representation was made to compare the IP plaque index and BOP probing bleeding between the subgroups diagnosed with periodontal disease (A, C) and the subgroups diagnosed with periodontal health (B, D) at the time of the initial consultation, using Welch’s statistical test.

**Figure 7:**
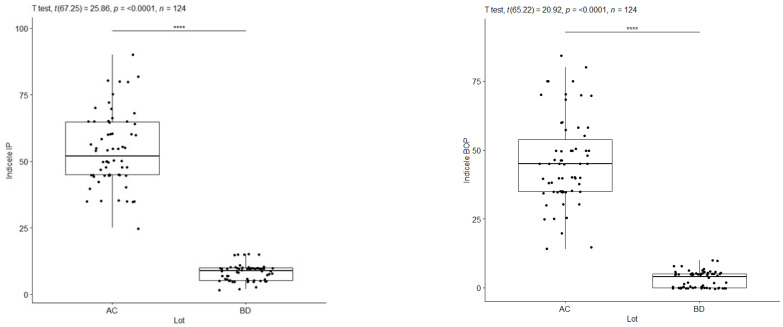
IP index—AC vs. BD at T1/BOP index—AC vs. BD at T1. ****: approx. 0, Extremely strong evidence against the null hypothesis.

Welch’s *t*-test for IP regarding pooled groups A and C versus B and D indicates that the alternative hypothesis is true, meaning that the difference in means between subgroup AC and subgroup BD is not equal to 0, with a range of 95% confidence, where the value of this difference is between 42.37 and 49.46, the t-statistic value is 25.864, the degree of freedom df = 67.254 and the *p*-value < 2.2 × 10^−16^ (approximately zero). The average values in the combined subgroups AC and BD are IP = 54.20% (for the subgroups diagnosed with periodontal damage) and, respectively, IP = 8.29% (for the subgroups with periodontal health). Welch’s test reveals significantly different IP index values between AC and BD sublots, graphically marked with four stars (****). Cohen’s d = 4.65 statistic also provides additional indications that these observed differences are large (Figure 7, Table 6 and Table 7).

**Figure 8:**
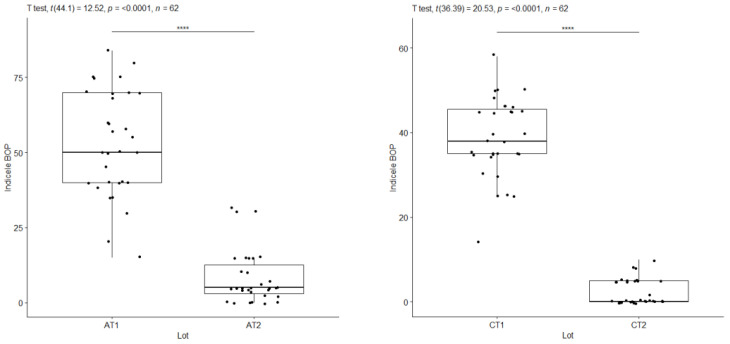
BOP index—A at T1 versus A at T2/IG index—C at T1 versus C at T2. ****: approx. 0, Extremely strong evidence against the null hypothesis.

Welch’s *t*-test, for BOP, at pooled subgroups A and C versus B and D shows that the alternative hypothesis is true, meaning that the difference in means for subgroups is not equal to 0, where subgroups AC = 45.93% for BD = 3.06%. Welch’s test reveals statistically significantly different values of the BOP index between AC and BD subgroups, graphically marked with four stars (****), at a 95% confidence interval, the value of the t statistic being 20.92, the degree of freedom df = 65.22, and the *p*-value < 2.2 × 10^−16^ (approximately zero). Cohen’s d statistic supports the previous statements, marking a high value of the difference between the groups, with a statistically significant difference and Cohen’s d = 3.76 (Figure 8, Table 8 and Table 9).

**Figure 9:**
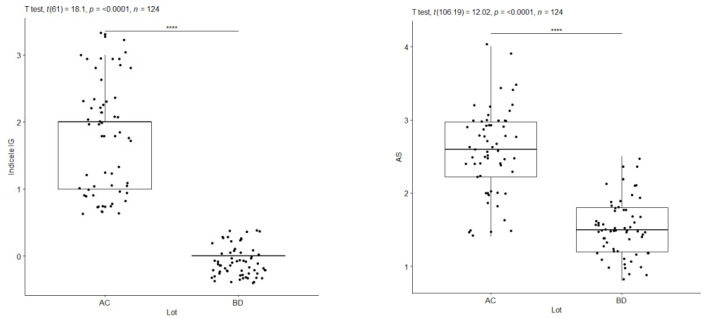
Index IG—AC vs. BD T1/AS—AC vs. BD at T1. ****: approx. 0, Extremely strong evidence against the null hypothesis.

**Figure 10:**
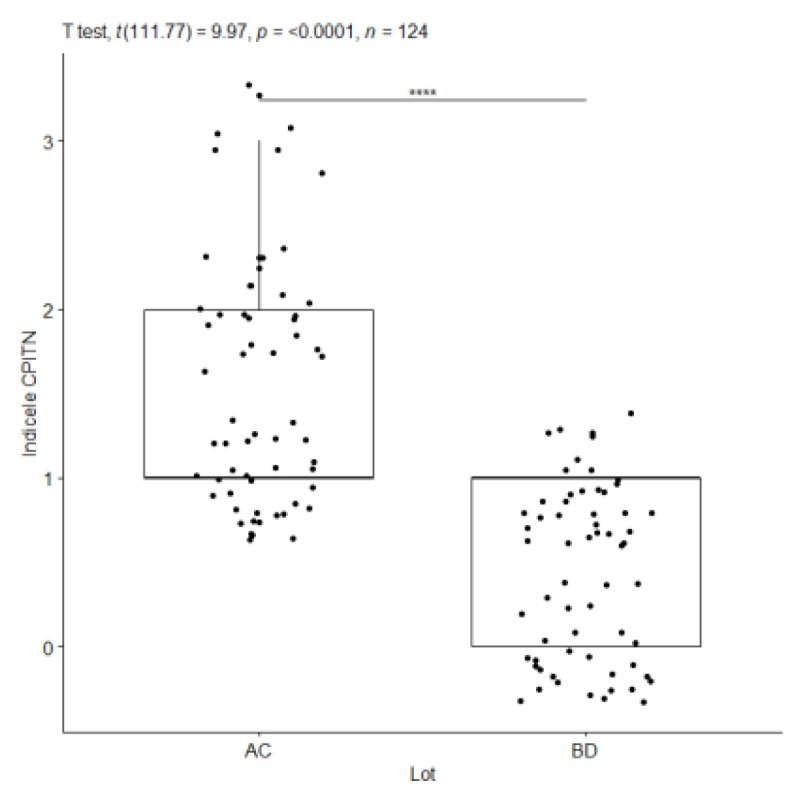
CPITN—AC vs. BD at T1. ****: approx. 0, Extremely strong evidence against the null hypothesis.

**Figure 11:**
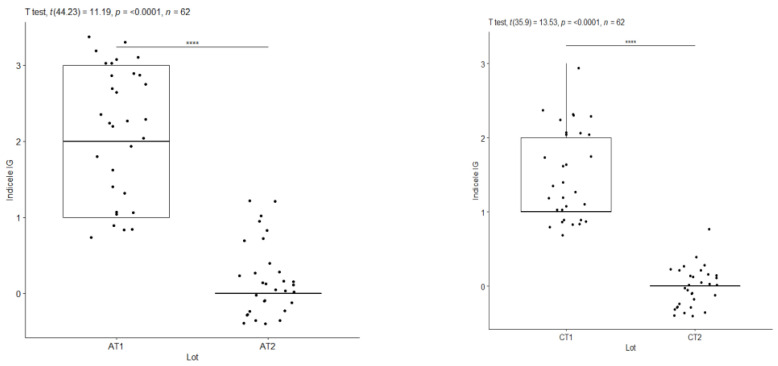
IG index—A at T1 versus A at T2/IG index—C at T1 versus C at T2. ****: approx. 0, Extremely strong evidence against the null hypothesis.

The statistical analysis shows a statistically significant difference between the gingival index (GI) values for the subgroups with periodontal damage compared to the subgroups without periodontal damage, a parameter measured at the time of the initial consultation. Analyzing probing depth, another extremely important parameter for the difference between health status and periodontal disease, the Welch test indicates that the alternative hypothesis is true, meaning that statistically significantly different values are present between the means of subgroups diagnosed with periodontal disease and of those diagnosed with periodontal health, the combined AC and BD subgroups, with a 95% confidence interval, for a value of this difference between 0.88 mm and 1.23 mm (Figure 9, Figure 10 and Figure 11).

For the CPITN, Welch’s *t*-test shows that the alternative hypothesis is true, meaning that the mean values are significantly different between the subgroups with a diagnosis of periodontal disease and those with a diagnosis of periodontal health, where the difference is not equal to 0, the 95% confidence interval is between 1.59 and 0.51, t is 9.97, the degree of freedom df = 111.77 and the *p*-value < 2.2 × 10^−16^. *p*-values indicate a significant difference, marked graphically with a number of four asterisks (****). Cohen’s d statistic supports the previous statements, marking a high value of the difference between the groups, where Cohen’s d = 1.79 (Figure 10).

To assess the evolution of gingival inflammation and the response to the applied treatment, as well as the influence of the general condition on the clinical parameters, the most representative indicators are GI and bleeding on probing (BOP). The values of these indices were compared for the subgroups with periodontal damage at the initial consultation (T1) and three months after treatment (T2). Comparison of these values revealed statistically significant differences influenced by cardiovascular disease.

As we observed from the previous data, the clinical parameters improved considerably at the level of groups with periodontal damage after the application of the treatment (Table 7, Table 8, Table 9, Table 10, Table 11, Table 12 and Table 13).

For subgroup A, 19 of the 31 patients progressed to periodontally healthy status; however, 12 patients retained periodontally affected status, 10 with localized forms and only 2 with forms of generalized gingivitis. For subgroup C, the changes were more obvious: the patients responded favorably to the applied treatment and, three months after the treatment, 28 of the 31 patients were diagnosed with periodontal health, and only 3 patients were diagnosed with localized forms of gingivitis.

Three months after treatment, a highly significant decrease in cases of periodontal damage was observed, and an improvement in the condition of periodontal tissues was observed, with a more obvious weight for patients in subgroup C, who did not present a diagnosis of affected general condition (Table 14).

## 4. Discussion

The presented study was carried out with the aim of highlighting the existing correlations between periodontal status, cardiovascular diagnosis, demographic data, oral hygiene status and periodontal clinical parameters, using applied and personalized analysis for the age range, full of changes, of 7–17 years. The study wanted to highlight the importance of maintaining good oral hygiene and, respectively, periodontal indices compatible with the state of periodontal health, especially for the group of patients with cardiovascular diseases, who present a high risk of infective endocarditis.

It was highlighted that, with age, child patients become more attentive and manage to achieve better oral hygiene, thus falling under the diagnosis of periodontal health. Between the four subgroups, no statistically significant difference was observed in terms of the average age of the included patients; however, the average age in the subgroups diagnosed with periodontal health is 15 years (under B) and 14 years (under D), unlike the subgroups with periodontal damage, where the average age was 13 years (under A) and 14 years (under C).

Looking at the gender distribution, we observed a statistically significant difference at the level of the subgroups with periodontal damage compared to the subgroups diagnosed with periodontal health. Thus, female patients were more concerned about oral health and the clinical parameters recorded were closer to the periodontal health status. Also, female patients wanted to participate in the study in a larger number than male patients (56 patients, representing 45.16%). In the subgroups with periodontal disease, subgroup A, C, the male gender predominated at 54.83% and 61.29%, compared to the subgroups without periodontal disease, subgroup B and D, where male patients represented 25.80% and 38.70%. The results obtained are in agreement with other studies carried out on various age ranges: patients with cardiovascular damage and periodontal disease present a greater number of male patients compared to the control group without periodontal damage or cardiovascular disease, where the female sex predominates [23,24,25,26,27].

Regarding the environment of origin for the whole lot, the most representative was the urban environment, it being much easier to reach for consultations and treatment, at 74.17% (number 90), and the value was only 26.82% (number 34) for the rural environment. The urban environment can represent a predisposing factor for the development of periodontal and cardiovascular diseases due to premature exposure to numerous risk factors. However, the collected data cannot support this hypothesis because the number of patients from rural areas examined was very small [26].

For vicious habits, smoking was confirmed as a predisposing factor for cardiovascular diseases and periodontal disease. At the level of subgroup A, the most smokers were identified, with a number of eight patients, representing 25.80%, compared to the rest of the subgroups, where the number of smokers was much smaller: at the level of subgroups B, D, a number of three (9.67%) smokers and two (6.45%) smokers were identified. Statistically significant differences were identified for the number of patients with vicious habits at the level of subgroups with periodontal disease compared to subgroups without periodontal disease. Smoking is a predisposing factor for cardiovascular diseases and periodontal disease, widely analyzed in the specialized literature, an aspect confirmed in the case of the present study [24].

Statistically significant differences were identified between subgroups for the heredo-collateral antecedents of periodontal diseases and cardiovascular diseases. This confirms the genetic predisposing factor for the development of periodontal diseases: the presence of certain structural characteristics that allow for the favorable development of different forms of periodontal disease, in the presence of the microbial determining factor, identified during the clinical examination for patients diagnosed with periodontal disease [27,28,29,30,31,32].

For the heredo-collateral antecedents of cardiovascular diseases, the presence of a larger number of relatives with an antecedent of cardiovascular diseases was observed at the level of the groups made up of patients who received a diagnosis of cardiovascular disease, subgroups A, B. There are statistically significant differences between the subgroups with cardiovascular diseases compared to the subgroups with good general condition regarding heredo-collateral antecedents. The genetic factor predisposing to cardiovascular diseases is confirmed, which is an observation that is consistent with other research from the specialized literature [27,28,29,30,31,32].

Statistically significant differences were observed for the values of the clinical parameters IP, BOP, IG, DP(AS) and CPITN, comparing the subgroups diagnosed with periodontal disease and the subgroups diagnosed with periodontal health. Also, statistically significantly different values were recorded between periodontal clinical parameters for subgroups A, C, diagnosed with periodontal damage, at the initial consultation T1 and at T2, three months after the application of the initial non-surgical periodontal treatment. The results are consistent with the results obtained by previous research [5,33,34,35].

In the case of patients from subgroup A, the maximum values and also the highest averages of the main clinical parameters were identified. It is confirmed that cardiovascular damage can cause the appearance of severe forms of periodontal disease for subgroup A, where maximum alarming values of the clinical indices were recorded (IP = 90%, BOP = 84%, IG = 3, DP = 4 mm and CPITN = 3), compared to sublot C, where IP = 70%, BOP = 58%, IG = 3, DP = 3.4 mm and CPITN = 2. The information obtained is consistent with previous research—for example, the study by Downing and colleagues, conducted between 2016 and 2019 in the United States—but contradicted by the research of Catekin and colleagues in 2015 in Turkey, which was a study that did not identify statistically significant differences regarding the state of oral health among children with cardiovascular diseases compared to the control group, children without cardiovascular diseases [5,35].

Cardiovascular diseases can represent a negative factor in the healing and favorable response to periodontal specialist treatment: patients in subgroup A found it more difficult to respond to the treatment applied than subgroup C. Three months after the application of periodontal treatment, changes in GI and BOP were observed, which were more statistically significant in the case of patients from subgroup C.

Cardiovascular diseases are a predisposing factor for gingival inflammation, in combination with the local microbial factor, an observation also confirmed by previous research [35]. After treatment, in the case of the group with a diagnosis of good general condition, the mean values of IG decreased almost to zero: IG = 0.03. The differences between subgroups A and C for the values recorded at T1 and T2 were statistically significant in the case of both subgroups, but more obvious for subgroup C, where patients who do not have a diagnosis of a general condition showed a much better response to the treatment applied.

Three months after treatment, a highly statistically significant decrease in the cases of periodontal damage was observed; however, they were not totally reduced: an improvement in the state of the periodontal tissues was observed with a more obvious weight for patients in subgroup C, who did not present a diagnosis of impaired general condition. The periodontal therapy performed in our study involved the application of a non-surgical therapeutic protocol in a single treatment step, so some periodontal sites could have remained partially loaded with periodontal pathogens. It is confirmed that a single treatment step is not enough, as it cannot eliminate all the affected sites influencing the host’s response to the applied therapy.

## 5. Conclusions

While most studies show a positive relationship between periodontal disease and cardiovascular diseases, analyzed in the case of adults, this study opens new perspectives, the results obtained in our study thus forming the basis of future research and confirming the need to substantiate and deepen the observations presented previously for children and teenagers.

Future studies should focus on promoting campaigns for the early diagnosis of periodontal diseases and measures for their treatment and prevention, especially in the case of patients with a compromised general condition.

The present study highlighted the interaction of three factors in the progression of periodontal diseases: subgingival microbiota, immune system response and environmental factors.

The systemic involvement of periodontal disease, studied in detail today, will be confirmed and clarified by future research and opens new horizons regarding a new approach to the control of cardiovascular diseases, with the inclusion of periodontal treatment as an independent preventive measure.

Considering the increased prevalence of periodontal disease, with early manifestation, in recent years, we emphasize a new responsibility of the general dentist and the periodontologist in the correct management of patients with premature periodontal disease or patients presenting certain local or general predisposing factors for the prevention of the development of systemic disorders related to the state of oral health.

## Figures and Tables

**Table 1:** Table regarding the values of *p*.

Meaning. Codes	*p*-Value	Interpretation
****	approx. 0	Extremely strong evidence against the null hypothesis
***	0 < *p* < 0.001	Very strong evidence against the null hypothesis
**	0.001 ≤ *p* < 0.01	Strong evidence against the null hypothesis
*	0.01 ≤ *p* < 0.05	Moderate evidence against the null hypothesis
.	0.05 ≤ *p* < 0.1	Weak evidence against the null hypothesis
ns	0.1 ≤ *p*	Not significant. Lack of evidence against the null hypothesis [20]

**Table 2:** Demographic data.

Sublots/Age Dates	Min.	1st Qu.	Median	Mean	3rd Qu.	Max.	Std. dev
Total lot	7.00	10.75	14.00	13.23	16.00	17.00	3.241271
Sublot A	7.00	10.50	13.00	13.03	16.00	17.00	3.219564
Sublot B	7.00	12.00	15.00	13.84	16.50	17.00	3.426337
Sublot C	7.00	10.00	14.00	13.10	16.00	17.00	3.187003
Sublot D	7.00	11.00	14.00	12.97	16.00	17.00	3.209194

**Table 3:** Distribution of vicious habits.

Vicious Habits		Total Lot		Sublot A		Sublot B		Sublot C		Sublot D
	No.	Dist.	No.	Dist.	No.	Dist.	No.	Dist.	No.	Dist.
Vicious habits Bruxism (B) Smoker (S) Or.respirator (OR)	1 (B) 0.80%		1 (B) 3.22%		0 (B)		0 (B)		0 (B)	
	21		11		3		4		2	
	15 (S) 12.96%		8 (S) 25.80%		3 (S) 9.67%		2 (S) 6.45%		2 (S) 6.45%	
	5 (OR) 4.03%		3 (OR) 9.67%		0 (OR)		2 (OR) 6.45%		0 (OR)	
Without vicious habits	103/83.06%		19/61.29%		28/90.32%		27/87.09%		29/93.54%	

**Table 4:** Genetic data.

Parameters	Total Lot	Sublot A	Sublot B	Sublot C	Sublot D
Family history of PD	DA = 44/35.48%	DA = 19/61.29%	DA = 6/19.35%	DA = 11/35.48%	DA = 8/25.80%
	NU = 80/64.51%	NU = 12/38.70%	NU = 25/80.64%	NU = 20/64.51%	NU = 23/74.19%
Family history of CD	DA = 36/29.03%	DA = 13/41.93%	DA = 11/35.48%	DA = 7/22.58%	DA = 5/16.12%
	NU = 88/70.96%	NU = 18/58.64%	NU = 20/64.51%	NU = 24/77.41%	NU = 26/83.87%

**Table 5:** Clinical parameters mean values per subgroups, min, max, standard deviation IP, BOP, IG, DP, CPITN, at the initial consultation and 3 months after treatment, for patients diagnosed with periodontal disease.

Indices/Parameters		T1 Sublot A				T2 Sublot A		
	Min	Max	Mean	Std. dev.	Min	Max	Mean	Std. dev.
IP%	25.00%	90.00%	57.48%	15.80%	3.00%	46.00%	16.03%	12.65%
BOP%	15.00%	84.00%	53.06%	17.84%	0.00%	32.00%	8.23%	8.91%
IG	1	3	2.13	0.85	0	1	0.23	0.43
DP	1.5	4	2.63	0.63	0	2.3	1.60	0.44
CPITN	1	3	1.90	0.75	0	2	0.58	0.62
Indices/parameters		T1 Sublot C				T2 Sublot C		
	Min	Max	Mean	Std. dev	Min	Max	Mean	Std. dev.
IP%	35.00%	70.00%	50.94%	10.30%	0.00%	15.00%	5.90%	3.51%
BOP%	14.00%	58.00%	38.81%	9.39%	0.00%	10.00%	2.35%	3.08%
IG	1	3	1.48	0.57	0	1	0.03	0.18
DP	1.4	3.4	2.55	0.53	0.8	2.9	1.70	0.45
CPITN	1	2	1.29	0.46	0	1	0.16	0.37
Indices/ parameters		T1 Sublot B				T1 Sublot D		
	Min	Max	Mean	Std. dev.	Min	Max	Mean	Std. dev.
IP%	5.00%	15.00%	8.87%	2.80%	2.00%	15.00%	7.71%	3.30%
BOP%	0.00%	10.00%	3.58%	3.07%	0.00%	10.00%	2.55%	2.78%
IG	0	0	0.00	0.00	0	0	0.00	0.00
DP	0.8	2.1	1.43	0.33	1	2.5	1.63	0.41
CPITN	0	1	0.58	0.50	0	1	0.45	0.51

**Table 6:** Welch test results: IP index—AC vs. BD at T1.

Index	Pair	Paired Differences Mean	Mean	Estimate Diff.	95% Conf. Interval Diff. Lower	Upper	t	df	*p* Value	P Signif.
IP	AC vs. BD (T1)	54.20%	8.29%	45.9	42.37	49.46	25.84	67.25	<2.2 × 10^−16^	large ****

****: approx. 0, Extremely strong evidence against the null hypothesis.

**Table 7:** Cohen’s d test results: IP—AC index vs. BD at T1.

Index	Pair	Cohen’s Effect Size	Magnitude
IP	AC vs. BD (T1)	4.65	large

**Table 8:** Welch test results: BOP index—AC versus BD at T1.

Index	Pair	Paired Differences Mean	Mean	Estimate Diff.	95%	Conf. Interval Diff.	t	df	*p* Value	P Signif.
					Lower	Upper				
IG	A (T1) vs. A (T2)	2.13	0.23	1.90	1.56	2.24	11.19	44.23	1.734 × 10^−14^	large ****
IG	C (T1) vs. C (T1)	1.48	0.03	1.45	1.23	1.66	13.52	35.90	1.161 × 10^−15^	large ****

****: approx. 0, Extremely strong evidence against the null hypothesis.

**Table 9:** Cohen’s d test results: BOP—AC index versus BD at T1.

Index	Pair	Cohen’s Effect Size	Magnitude
BOP	AC vs. BD (T1)	3.76	large

**Table 10:** Welch test results: IG index—A at T1 vs. A at T2/IG index—C at T1 vs. C at T2.

Index	Pair	Paired Differences Mean	Mean	Estimate Diff.	95%	Conf. Interval Diff.	t	df	*p* Value	P Signif.
					Lower	Upper				
IG	A (T1) vs. A (T2)	2.13	0.23	1.90	1.56	2.24	11.19	44.23	1.734 × 10^−14^	large ****
IG	C (T1) vs. C (T1)	1.48	0.03	1.45	1.23	1.66	13.52	35.90	1.161 × 10^−15^	large ****

****: approx. 0, Extremely strong evidence against the null hypothesis.

**Table 11:** Cohen’s d test results: IG index—A at T1 vs. A at T2/IG index—C at T1 vs. C at T2.

Index	Pair	Cohen’s Effect Size	Magnitude
IG	A (T1) vs. A (T2)	2.84	large
IG	C (T1) vs. C (T2)	3.44	large

**Table 12:** Welch Test Results BOP Index—A at T1 vs. A at T2/BOP Index—C at T1 vs. C at T2.

Index	Pair	Paired		Estimate Diff.	95%	Conf. Interval Diff.	t	df	*p* Value	P Signif.
		Differences Mean	Mean		Lower	Upper				
BOP	A (T1) vs. A (T2)	53.1%	8.23%	44.8	37.62	52.05	12.52	44.102	4.077 × 10^−16^	large ****
BOP	C (T1) vs. C (T1)	38.80%	2.35%	36.5	32.85	40.05	20.53	36.38	<2.2 × 10^−16^	large ****

****: approx. 0, Extremely strong evidence against the null hypothesis.

**Table 13:** Cohen’s d test results: BOP index—A at T1 vs. A at T2/BOP index—C at T1 vs. C at T2.

Index	Pair	Cohen’s Effect Size	Magnitude
BOP	A (T1) vs. A (T2)	3.18	large
BOP	C (T1) vs. C (T2)	5.21	large

**Table 14:** Periodontal diagnosis three months after treatment T2 compared to T1.

Periodontal Diagnosis T2/T1	Number Total Lot		(%)		Number sb. A		(%) sb. A		Number sb. C		(%) sb. C	
	T2	T1	T2	T1	T2	T1	T2	T1	T2	T1	T2	T1
Generalized biofilm-induced gingivitis	0	6	0	4.83	0	5	0	16.12	0	1	0.00	3.22
Generalized biofilm-induced gingivitis associated with vicious habits	2	14	1.61	11.29	2	10	6.46	32.25	0	4	0.00	12.90
Hormonally associated generalized biofilm-induced gingivitis (phen. hypergrowth)	0	1	0	0.80	0	1	0	3.23	0	0	0.00	0.00
Generalized biofilm-induced gingivitis associated with local condition—dental eruption	0	3	0	2.41	0	2	0	6.45	0	1	0.00	3.22
Generalized biofilm-induced gingivitis complicated with hyperplastic phenomena	0	2	0	1.61	0	2	0	6.45	0	0	0.00	0.00
Localized biofilm-induced gingivitis	9	36	7.25	29.03	6	11	19.35	35.48	3	25	9.68	80.65
Localized biofilm-induced gingivitis associated with vicious habits	3	0	2.41	0.00	3	0	9.64	0.00	0	0	0.00	0.00
Localized biofilm-induced gingivitis associated with local condition—tooth eruption	1	0	0.81	0.00	1	0	3.22	0.00	0	0	0.00	0.00
Periodontal health	109	62	87.90	50.00	19	0	61.29	0.00	28	0	90.32	0.00
TOTAL	124	124	100%	100%	31	31	100%	100%	31	31	100%	100%

## Data Availability

The data presented in this study are available upon request from the corresponding author. The data are not publicly available due to privacy reasons.
